# A Systematic Mapping Study on Integration Proposals of the Personas Technique in Agile Methodologies

**DOI:** 10.3390/s21186298

**Published:** 2021-09-20

**Authors:** Patricia Losana, John W. Castro, Xavier Ferre, Elena Villalba-Mora, Silvia T. Acuña

**Affiliations:** 1 Escuela Politécnica Superior, Universidad Autónoma de Madrid (UAM), 28049 Madrid, Spain; patricia.losana@estudiante.uam.es (P.L.); silvia.acunna@uam.es (S.T.A.); 2 Departamento de Ingeniería Informática y Ciencias de la Computación, Universidad de Atacama (UDA), Copiapó 1530000, Chile; john.castro@uda.cl; 3 Center for Biomedical Technology (CBT), Universidad Politécnica de Madrid (UPM), Pozuelo de Alarcón, 28223 Madrid, Spain; xavier.ferre@upm.es; 4 Centro de Investigación Biomédica en Red en Bioingeniería, Biomateriales y Nanomedicina (CIBER-BBN), 28029 Madrid, Spain

**Keywords:** personas, user profiling, human–computer interaction, user-centered design, agile methodology, software engineering, systematic mapping study

## Abstract

Agile development processes are increasing their consideration of usability by integrating various user-centered design techniques throughout development. One such technique is Personas, which proposes the creation of fictitious users with real preferences to drive application design. Since applying this technique conflicts with the time constraints of agile development, Personas has been adapted over the years. Our objective is to determine the adoption level and type of integration, as well as to propose improvements to the Personas technique for agile development. A systematic mapping study was performed, retrieving 28 articles grouped by agile methodology type. We found some common integration strategies regardless of the specific agile approach, along with some frequent problems, mainly related to Persona modelling and context representation. Based on these limitations, we propose an adaptation to the technique in order to reduce the creation time for a preliminary persona. The number of publications dealing with Personas and agile development is increasing, which reveals a growing interest in the application of this technique to develop usable agile software.

## 1. Introduction

Usability is a characteristic of software quality used in most classifications [1,2], which must be addressed throughout the entire interactive software development process [3,4]. In order to develop a usable software system, it is necessary to understand the users for whom the system is destined [5,6]. There are myriad techniques within the human–computer interaction (HCI) discipline to perform a user analysis, namely, studying and modeling the person that will use the software system, one of which is the Personas technique [7]. It consists of designing a user model from data obtained through interviews with real users, as well as guiding the application design with the users’ preferences and avoiding the creation of a design by developers based solely on their own assumptions. In this paper we capitalize the term ‘Personas’ when we refer to the technique and use lowercase ‘persona’ when we refer to the representation of a specific user type.

The Personas technique, described by Alan Cooper [8], is a user-centered design (UCD) tool that seeks to conceptualize the behavior of real users within user models, with the objective of improving the usability of the design. In this way, although a persona is fictitious, the objectives it addresses are real since they are synthesized from the observations of end users. The technique enables the design and development teams to empathize more easily with user preferences [9]. Personas is complementary to other user research techniques. While discussion with real users is a good method to generate empathy in the design team for the user viewpoint, Personas can help provide a practical foundation for such discussions. The technique enables designers to identify the key points that define diverse users and their goals, expressed in a way that results in user-centered design decisions.

Agile development has a strong focus on providing value to the customer. While XP includes an ‘on-site customer’ as one of its practices, Scrum has a ‘product owner’. These customer representatives allegedly bring the preferences and viewpoints of the end user to the table. However, the customer and the user are different people in bespoke systems, since the customer is the one actually paying for the software while the user is the person who will use the system when it is deployed. The customer and the user are the same only for market-aimed products. Personas can help avoid these misunderstandings by enabling the agile development team to adopt the user viewpoint.

The Personas technique was systematized to the same level as Software Engineering (SE) techniques through the work of [10,11]. Later, in the study performed by [12], the technique was adapted for integration within an agile development process and was then evaluated through a case study by [13], which facilitated testing the viability and impact of applying the Personas technique within a real agile project. This systematization of the Personas technique has been validated by its application in four different case studies [11,12,13,14].

The next step in this line of research corresponds to studying the state of the art of incorporating the Personas technique within agile processes in order to establish how this is being used within agile projects and to identify potential improvements for the technique. Although there have been other systematic studies related to the integration of UCD within agile software development processes (ASDPs) [15,16,17,18,19], they focus on the problems of integration rather than on addressing the integration strategies of the techniques, and none of them focus specifically on Personas. Since Personas was found to be the most commonly used technique for usability adoption in ASDPs [20], we deem it necessary to investigate the different integration strategies that have been adopted. To this end, our study aims to propose an adaptation to the Personas technique by identifying the different approaches to its integration through a literature review carried out by means of a systematic mapping study (SMS). The results of the SMS are reported in this study.

Paper organization. In Section 2, we present the state of the art of the integration of HCI techniques into ASDPs and Personas in particular. In Section 3, we describe the research method of the SMS. In Section 4, we discuss the results of the SMS. Section 5 presents possible threats to validity and, finally, the conclusions are presented in Section 6.

## 2. State of the Art

Although there are different agile methodologies, all of them are characterized by being iterative, promoting developer–client collaboration, and receiving feedback from the client throughout the development life cycle. The most relevant methodologies are: Dynamic Systems Development (DSDM) [21], Functionality Driven Development (FDD) [22], Lean Software Development [23], Scrum [24] and eXtreme Programming (XP) [25]. Agile philosophy is characterized by evaluating the functionality of prototypes with users over short iterations to identify possible discrepancies between customer needs and design decisions. Accordingly, usability should be an important characteristic of quality in agile development, to ensure that user needs are adequately addressed. In order to develop usable software and prevent disuse, the integration of UCD techniques within agile methodologies has increased [26,27].

The integration of UCD into agile approaches has traditionally encountered obstacles related to the lack of usability awareness and the different foci of HCI techniques and common agile activities. The agile manifesto focuses on providing value for the customer in the form of functioning software, whereas UCD requires extensive user research activities that can be regarded as an up-front period of investigation that delays the actual writing of code [28]. Both UCD and agile approaches identify the need for an iterative process that can handle uncertainty. However, the need to incorporate UCD activities into the overall agile process remains a challenge [29].

Even though Personas emerged in the HCI field and not in agile methodologies, the technique has been sought out for use in agile processes in order to help development teams produce a better design [30].

Among the different ways of achieving the integration of UCD into agile processes, an agile version of Personas stands out, consisting of a partial application of the technique at the beginning of the development, followed by refinement and completion throughout the iterations. This agilized version of Personas helps to overcome the time constraints that exist in the agile development process [31,32].

Various examples have been found in the agile literature that prove the Personas technique helps to improve the usability of interfaces and to meet user requirements during the agile lifecycle [33,34,35]. It is a useful tool for mediating communication between developers and designers, measuring design effectiveness, and determining how a product should behave.

Several systematic reviews have been carried out to study the integration of UCD and agile development [16,17,18], the artifacts used for such integration [19], and proposals for integrated approaches which are grouped under the user-centered agile software development term [15].

Sohaib and Khan present the conflicting visions of both approaches [17]. They mention ‘extreme personas’ as a proposal in one of the studies but do not describe the methodological approach followed for their review, thus limiting its validity from a scientific point of view.

Silva et al. claim that a common process model for integration of UCD in agile development underlies the different approaches to such integration, as identified in their systematic review [16]. Around eight of their 58 selected papers address Personas (no precise figure is included, just a bar diagram), and it is identified as a technique used for design.

Salah et al. identify the challenges for UCD and agile integration in their systematic review [18]. Personas is mentioned as a successful practice for addressing the problem of a lack of documentation.

Garcia et al. [19] study the artifacts used for communication between the UCD and agile teams in their systematic mapping study and identify Personas as one of the most cited artifacts (15 citations in their 56 selected publications).

Brhel et al. carry out a literature review about the principles constituting a user-centered agile software development approach, identifying that 15% of the 83 papers selected mentioned the Personas technique [15].

These existing literature reviews identify Personas as a useful tool for the integration of UCD into agile development, but they do not provide any detail about how to achieve such integration. Therefore, the next step would be to discover how such a technique is being integrated in the various types of agile development processes to achieve an effective usability result in the software product, and to learn from the experience of different authors, with the aim of compiling a set of integration recommendations.

## 3. Research Method

The secondary study presented in this paper has been developed following the guidelines established by Kitchenham et al. for conducting an SMS in the field of SE [36,37]. Following these guidelines, the activities we carried out were: (i) formulating the research questions, (ii) defining the search strategies, (iii) selecting the primary studies, (iv) extracting the data, and (v) synthesizing the extracted data. The information extracted from the primary studies should be consistent with the research questions and the response should highlight the similarities and differences between the research results to facilitate further analysis.

### 3.1. Research Questions

The information extracted from the primary studies aims to answer the following research questions: (RQ1) What is the state of the art regarding the integration of the Personas technique in agile processes? (RQ2) What are the main ways of integrating the Personas technique in agile software development? (RQ3) What are the main limitations of integrating the Personas technique in agile software development and what improvements can be introduced to overcome the limitations?

### 3.2. Define the Search Strategy

The SMS begins with the identification of the keywords, which are those that appear most frequently in the control group (CG) articles: a reduced set of 13 papers [26,27,28,30,31,32,33,34,38,39,40,41,42]. For the CG we selected articles which were directly related to the usage, application, or integration of Personas in agile development projects. A complete list of the CG articles can be found in Appendix A.

In order to assess the validity of the search strings formed, we checked the number of CG articles retrieved within the Scopus database. We considered that, being the largest database [43], Scopus was where the highest number of CG articles would be found; therefore, the search string that retrieved the highest number of results from the CG would be the most suitable one to use for our search.

To obtain the keywords, a table was generated with the frequency of all the words and combinations of words that appeared in the CG articles, with the help of the Atlas.ti 9 software [44]. We selected only those words directly related to the research questions and that were present in a significant percentage of the CG articles. Subsequently, each one of the words obtained was assigned a value from 0 to 1, determined by its frequency of use, so that the word most frequently repeated in the various CG articles had the value 1. Table 1 shows a fragment of the list of words obtained as a result of this selection process. It shows the words, the percentage of CG studies it appeared in (coverage), the frequency of its appearance throughout the CG studies, and its assigned weight, based on the two previous columns. The complete list can be found in Appendix B.

### 3.3. Formation of the Search String

Once the keywords were identified, several search strings were constructed. For their construction, the words were grouped into synonyms of different components: words related to (i) the Personas technique, (ii) usability, (iii) integration, and (iv) agile processes. The logical operator AND was used to join each of these components, while the logical operator OR was used to include synonyms of words from the same component. A total of three search strings were constructed, as shown in Table 2. For each of the strings, the terms that are different appear in bold type and the number of CG studies retrieved from the Scopus database was recorded.

The Scopus database contains 11 of the 13 papers from the CG, omitting papers [26] and [34]. The three generated strings were tested in the Scopus database, and we then selected the one that retrieved the largest quantity of CG articles. The structure of the final search string is shown in Table 3.

Although the search string tests were performed in Scopus, the largest database of peer-reviewed literature [43], the searches were also performed in the ACM Digital Library and IEEE Xplore in order to acquire more results. No date limit was used in order to cover all studies published up to the date of the search (December 2020). The databases were analyzed sequentially, using the search fields shown in Table 4. If a duplicate appeared, the first result was kept.

### 3.4. Selection Criteria

The criteria used to select the primary studies are summarized below.

- Inclusion criteria: the paper “is directly related to the use of the” OR “describes the application of” OR “integrates the” Personas technique in agile software development AND “is published in journals OR conference proceedings OR book chapters”.
- Exclusion criteria: the paper “is a systematic literature review” OR “is a systematic mapping study” OR “is an SMS” OR “is a primary study but the topic is not directly related to integration or the use of Personas in agile software development” OR “is not written in English”.

### 3.5. Select the Studies

A total of 104 papers from 2003 to 2020 were found in the different databases. After excluding duplicate articles, the number was reduced to 78. Next, a peer review was carried out on these articles, applying the selection criteria to the title and abstract. The peer review team consisted of two authors of the paper who are experts in the HCI Personas technique and agile processes. The lead role was taken by the student, since the review formed part of her master’s thesis. Table 5 shows the percentage agreement [45] and the Cohen’s Kappa coefficient (k) [46] between the researchers. The reviewers agreed on 97 out of 104 studies, which is considered an almost perfect agreement. As for the Kappa coefficient, we obtained k = 0.86, which, according to [47], is indicative of a substantial agreement.

**Figure 1:**
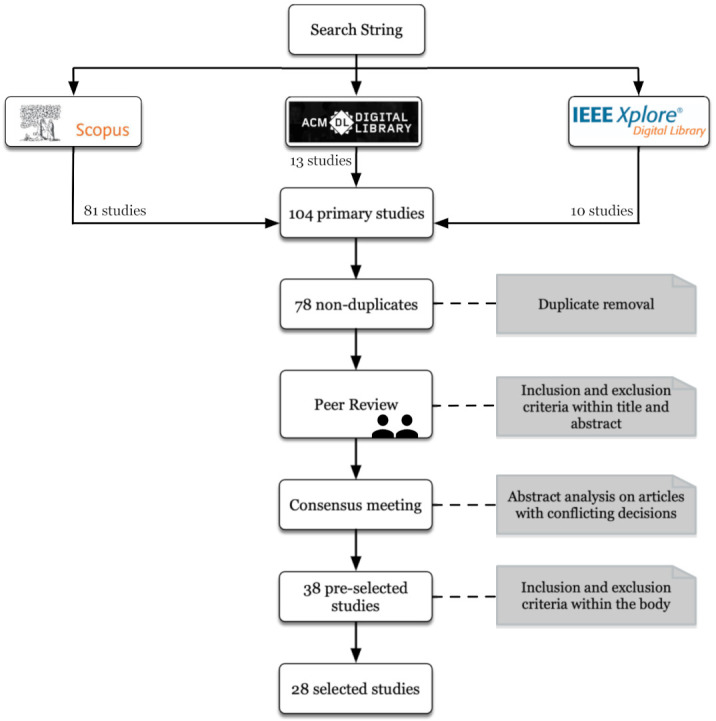
Steps followed during the systematic mapping study.

The selected articles were validated during a consensus meeting, in which we analyzed the abstracts of articles with conflicting decisions, thus reducing the total to 38 pre-selected articles. During this consensus meeting, a third researcher, who is also an expert in the field and an author of the paper, mediated the final decision in cases of divergence among the review team. After the meeting, the selection criteria were again applied to the full text of the remaining articles. Figure 1 shows the entire filtering and analysis process with the inclusion and exclusion criteria used to select a total of 28 papers [27,31,32,33,39,40,48,49,50,51,52,53,54,55,56,57,58,59,60,61,62,63,64,65,66,67,68,69]. A complete list of the primary studies can be found in Appendix C.

The results of applying the different filters during the selection process for each of the databases can be seen in Table 6.

## 4. Results and Discussion

### 4.1. State of the Art of the Personas Technique Integration

**Figure 2:**
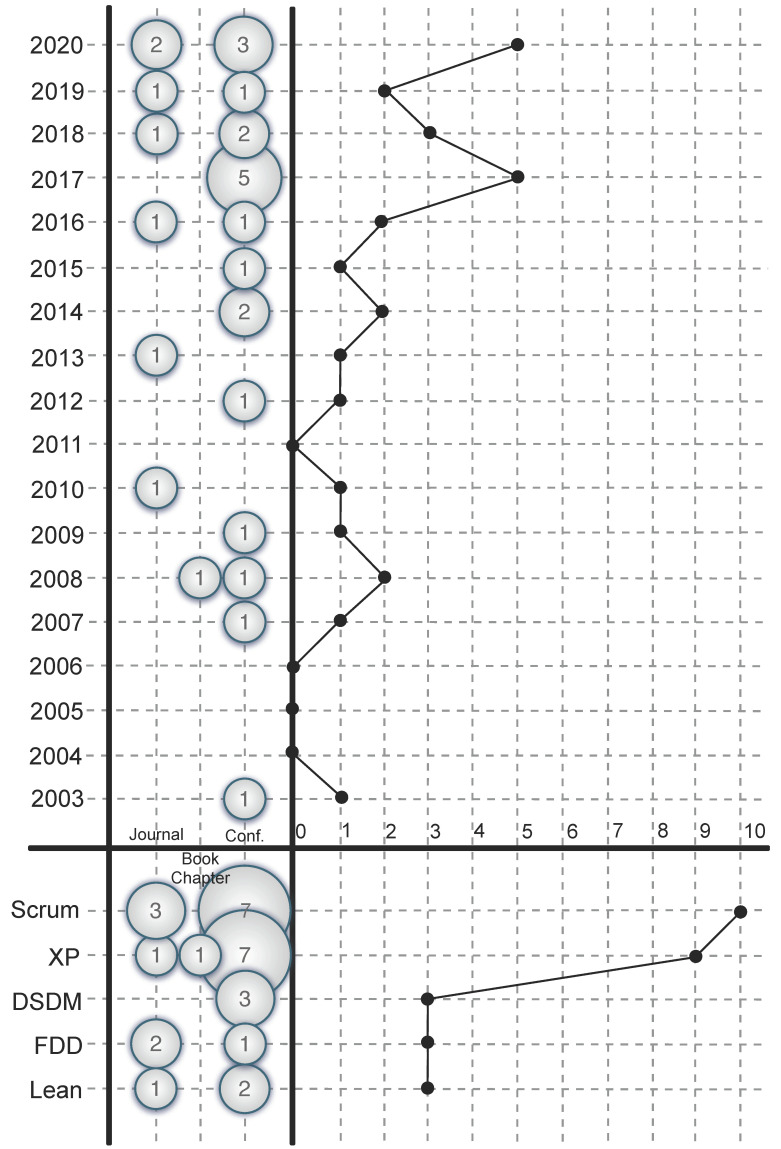
Mapping for the primary study distribution between the different agile process categories along the year and type of publication (answer to RQ1).

To assess the state of the art of the integration of the Personas technique in agile processes, each of the 28 selected studies was classified according to the type of agile process used. Figure 2 synthesizes the results using two bubble scatter plots. The upper graph represents the number of articles published per year, according to the type of publication (conference, journal, or book chapter). Similarly, the lower graph plots the type of publication against the agile methodology in which the Personas technique has been integrated. Thus, the bubbles are located at the intersections between the two axes and their size is proportional to the number of publications for each combination of values.

Although there have been studies on integrating the Personas technique in agile processes since 2003, the interest in its integration in agile development has been increasing since 2016. In addition, most primary studies have focused on Scrum and XP agile processes (see bottom part of Figure 2), and have been published in specialized conferences and journals, suggesting that the interest of the scientific community in integrating this technique in agile processes is on the rise.

### 4.2. Main Ways of Integrating the Personas Technique

We identified and extracted: (1) the main forms of integration of the Personas technique in agile software development for the selected articles; (2) the description of how this integration was carried out; and (3) the life cycle activity in which it was integrated.

Table 7 shows a synthesis of the different forms of integration for each type of agile process, based on the selected articles. For each type of agile process, the most commonly used methods of integration found in the different studies are listed, sorted by life cycle activity. Details of the integration proposed in each individual study is contained in Appendix D.

#### 4.2.1. Scrum

The studies that integrated Personas into Scrum proposed holding creative team sessions prior to the start of development to complete the personas narratives. There are several studies which conducted a brainstorming session with students [51,52,53], where they completed the personas narratives with previously generated sentences, and later associated them with the most convenient user stories [54]. The study by the authors of [55] proposes using mind maps to connect the different personas. Studies [39,56,57] associate each persona with a specific context, a short description of preferences, and a motivation, which makes it easier for developers to empathize with end users during development. All of these studies address user goals in incremental iterations, validating the functionality of the goals with users after each iteration.

Moreover, in [58,59] the authors include non-functional requirements as goals as well, in order to obtain high-fidelity prototypes.

#### 4.2.2. XP

The studies dealing with the integration of Personas in XP interview users and investigate their context in order to empathize more easily with them, thus orienting development to their preferences [31,32,33,40,66,67,68]. Furthermore, the authors of [27,33] propose an iterative refinement during which they collect user information in parallel with coding activities. This approach allows that, every time the team receives new information from users, the existing personas will be refactored, and new personas will be created in case they are needed to fit the new user requirements. In study [69] the authors propose designing a mind map in a similar way to the Scrum study [55] mentioned above, with the aim of connecting what the persona wants and how he/she wants it, using colors to highlight what is most relevant.

#### 4.2.3. DSDM

The studies conducted on DSDM agile processes create the persona models using both an interview and an analysis of the user stories. In the case of [48], instead of a narrative, they create drawn sketches of personas based on the information obtained in the interviews. In [49], a preliminary design thinking session is carried out in which user stories are analyzed by all team members. In both cases, the technique is integrated during the elicitation and requirements analysis activity. The authors in study [50] also integrate the technique during the planning and design activity. All three studies validate the assignment of personas to user stories with end users before starting the design. Moreover, they all validate the solutions after elaborating on their designs.

#### 4.2.4. FDD

The studies that integrated Personas in FDD analyze the interactions of people to establish behavioral patterns. The authors in studies [60,61] abstract patterns from user stories and assign them to specific subjects. In [62], they conduct further interviews involving emotional analysis experts, in order to more easily identify end user personalities.

#### 4.2.5. Lean

The integration of Personas in the Lean studies analyzed start by knowing the user groups targeted by development, either through questionnaires [63] or contextual investigations [64]. In [65], they group the results into clusters of users based on the preferences and behaviors found, customizing subsequent designs according to the patterns found in each cluster.

### 4.3. Main Limitations of Personas Integration

Throughout the literature review, two main limitations were encountered when integrating the Personas technique in agile software development. The first limitation, considered the most relevant, is determining the appropriate amount of necessary and sufficient information that should appear in the initial description of the persona. It should be detailed enough for the development team to empathize with the user’s needs, but not so detailed as to threaten the time restrictions of an agile process [50,69]. An interesting solution could be to create the initial models of personas from templates with predefined phrases, as proposed in [50,52]. Although the personas created by self-reported information during interviews might not be reliable [69], an analysis of the primary studies suggests that this could become a standardized aspect of integrating the Personas technique with agile methodologies. This way, the first persona model would be created with a much lower temporal impact on the project. The first persona sketch would be simple, but it would be refined throughout the iterations, as reported in studies [31,40,60,64,68].

The second limitation shared by different studies is representing the context in which a user persona wants to perform an action, and the possible interaction with other personas within the same requirement [32,39,40,50,56,60,63,65,67,69]. Context issues can emerge due to the fact that personas are created independently of each other, with the purpose of solving specific existing use cases, but without accounting for how they interact [48,50,56].

Amongst all the studies that share this limitation, only one study [67] proposes a solution: to design an entity-relationship model to allow the differentiating of the relationships between different personas and their user stories.

Within the model there would be three entities: User Story, Persona, and Navigation Relationship. The User Story entity would have a user value attribute, with the objective of prioritizing the list of requirements. The Persona entity would contain attributes related to the context of use, so that it would be possible to differentiate between different types of requirements according to the user. Finally, the Navigation Relationship entity would include attributes representing the interactions between Persona and User Stories, thus allowing different contexts of use between different Persona entities for the same User Story and, therefore, representation of more complex usage scenarios.

**Figure 3:**
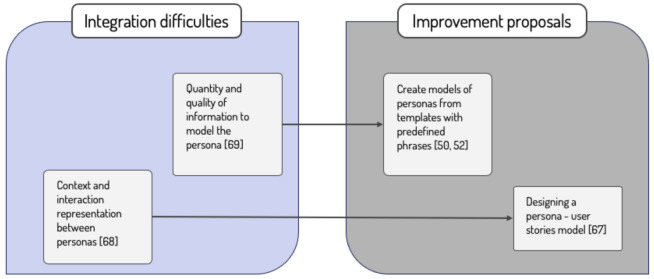
Integration limitations and proposed improvements (answer to RQ3).

A synthesis of the results obtained for RQ3 is shown in Figure 3.

In addition to the limitations found, the inclusion of Personas resulted in better SE results. The authors of study [69] present a comparison between using or not using Personas throughout software development by means of a quality checklist. In our study, we found that understanding of the users and empathy towards them increases from 30% when only using user stories, to 100% when using the Personas technique. As for relationships among users, the use of Personas increases the knowledge from 28% to 57%.

### 4.4. Adaptation Proposal for the Personas Technique to Scrum

Based on the proposal to create initial models from templates [50,52], we propose an agilization to Cooper’s Personas technique that focuses on automating the process of finding patterns among users during an initial contextual investigation, thus reducing the temporal impact on the agile project.

Automation could be achieved by using an automatic synthesizer of responses to the questionnaire, such as the one provided by the Google Forms software. This synthesis would allow a visual and immediate identification of the main patterns of responses to each question, using the interview itself as a template for the creation of the initial personas. These models would be created with a very low temporal impact, and would be refined at each iteration, thereby distributing the temporal impact of the technique along the development’s lifetime. For the process to be agile, it is critical that the questions and answers designed in the interview provide meaningful information.

In order to test the usefulness of this proposed adaptation of the Personas technique for Scrum, we intend to validate it through multiple case studies.

## 5. Validity Threats

Throughout this study, we have assessed certain aspects that could jeopardize the validity of the study. The main threat to validity is the possibility of bias in the selection process of the primary studies. To reduce this bias, we followed the guidelines proposed in [36,37], as well as the validity checklist proposed in [70].

In order to ensure the validity of the study selection during the search process, several considerations were made. The SMS was carried out using the three most relevant databases for the purpose of this search: Scopus, ACM, and IEEE Xplore. This approach ensured the identification of the most relevant publications in the field from a variety of journals and conferences. However, if additional databases had been included, new results and complementary information could have been obtained.

Regarding the construction and adequacy of the search string, we reduced the risk of not including relevant search terms by creating three search strings, constructed from the most common relevant words appearing in a selected group of 13 key papers (the CG). Search tests were carried out to select the final search string, checking that the maximum possible number of papers from the CG were returned. as John W. Castro and Silvia T. Acuña are experts in the Personas technique and in integrating usability activities in agile processes. They selected the CG papers to provide the best answers to the research questions, based on their previous research activities in this area. A validity threat could be that the criteria for the selection of these CG articles is not the most appropriate. In order to minimize this threat, we based the selection criteria on the research questions established for our study.

Regarding access to the content, researchers could access the full text of all primary studies that passed the pre-selection criteria, so there were no selected studies that lacked the full text. Another threat to the study validity is the application of the selection criteria and analysis of abstracts of the articles found. In order to minimize subjectivity, the selection process was carried out in parallel by two members of the research team, with usage of the Cohen’s Kappa statistics to evaluate disagreements between them. The selected articles were subsequently agreed upon in a group meeting. For duplicate articles, the selection strategy was to keep the first result.

With regards to validity of the data, the analysis was carried out on a sample of 28 primary studies. Synthesis and data extraction were performed on these studies to look for possible relationships in the integration processes, using the different types of agile methodology as a consensus classification criterion.

## 6. Conclusions

Throughout this work, we have presented a systematic mapping study on the integration of the Personas technique across different types of agile processes, with the objective of understanding the current state of the art of its integration, and to establish a knowledge base that would allow proposing future improvements to the technique. The study started by identifying keywords in a set of articles called the control group. These keywords were combined to formulate a search string that allowed us to carry out an in-depth analysis of all primary studies related to the integration of both concepts (the Personas technique and agile methodologies) across different databases. Subsequently, we applied a selection criterion to exclude those publications that did not contain information to answer the research questions. From the in-depth analysis of these 28 studies, it has been possible to see that the integration of the Personas technique in agile development has been increasing since 2016, which reflects a growing interest of the scientific community in the field, especially in the agile processes of Scrum and XP.

After synthesizing the results from various publications, we have observed that, regardless of the type of agile process in which the Personas technique was integrated, there were some common aspects among them. On the one hand, integration always takes place during the activity of elicitation and requirements analysis, and may involve other activities of the software development process. Additionally, the first integration step always consists of an analysis of the target users, either by questionnaires, interviews, or brainstorming. This step enables the acquisition of an initial persona model that can be refined or adapted according to new user requests that arise with each iteration. On the other hand, the main limitations in integrating this technique within agile methodology relate to the need to invest time in defining the persona, a crucial part of the paradigm user-centered design, in which usability and detailed knowledge of the end user is a priority. In contrast, the objective in agile development is to cover functionalities from early iterations with value for the client, which may reduce the time that is needed for the design.

Since most of the integration problems were related to the temporal impact of the application of the Personas technique, we propose a simplification of the initial contextual research. The proposal begins with a contextual investigation, where the personalities and environments of the users are studied by means of an interview. The interview should have questions that directly correlate with the behavioral variables of interest, and each answer option should be a possible range.

Our results provide support for the development and application of this technology in the future, aiming to obtain agile development processes with increasingly user-centered results. Future work will attempt to validate the proposal of this more agile technique in which the initial model of the personas is obtained directly from a user questionnaire and is then refined throughout the following iterations. This proposal, which distributes the time dedicated to the technique among the iterations, will be validated in a later case study. Additionally, we plan to carry out an experimental validation of the proposed adapted Personas technique through multiple case studies.

## Figures and Tables

**Table 1:** Fragment of the list of words obtained from the selection process.

Words	Coverage (%)	Frequency	Weight
Agile	100	630	1
User	100	613	0.987
Usability	100	578	0.923
Product	100	225	0.684
Personas	92.86	169	0.602
Interaction	92.86	148	0.585
eXtreme Programming	85.71	53	0.472

**Table 2:** Search strings.

ID	Search String	Studies Found	GC Found
1	Personas AND (usability OR user OR UCD OR “user-centered design” OR UX OR “user experience” OR HCI OR “interface design” OR “interaction design”) **AND integrating** AND (agile OR “agile development” OR “extreme programming” OR Scrum OR sprint OR “agile method” OR “agile software development” OR “agile process”)	4	0
2	Personas AND (usability OR user OR UCD OR “user-centered design” OR UX OR “user experience” OR HCI OR “interface design” OR “interaction design”) AND (agile OR “agile development” OR “extreme programming” OR Scrum OR sprint OR “agile method” OR “agile software development” OR “agile process”)	69	8
3	Personas AND (usability OR user OR UCD OR “user-centered design” OR UX OR “user experience” OR HCI OR “interface design” OR “interaction design”) AND (agile OR “agile development” OR “extreme programming” OR Scrum OR sprint **OR “user stories”** OR “agile method” OR “agile software development” OR “agile process”)	81	9

**Table 3:** Final search string.

Keywords				
		usability OR		agile OR
		user OR		“agile development” OR
		UCD OR		“extreme programming” OR
		“user-centered design” OR		Scrum OR
Personas	AND	UX OR	AND	sprint OR
		“user experience” OR		“user stories” OR
		HCI OR		“agile method” OR
		“interface design” OR		“agile software development” OR
		“interaction design”		“agile process”

**Table 4:** Search field per database.

Database	Search Fields	Number of Results
Scopus	“Title OR Abstract OR Keywords”	81
ACM Digital Library	“Abstract OR Title”	13
IEEE Xplore	“Title OR Abstract”	10

**Table 5:** Agreement matrix for nominal variable.

Agreement Matrix for Nominal Variable			
Researcher 1		Researcher 2	
		Accepted	Rejected
	Accepted	38	6
	Rejected	1	59
	Total	39	65

**Table 6:** Number of remaining studies after filtering the database results.

Database	Studies Found	Duplicate-Free	Pre-Selected Studies	Primary Studies
Scopus	80	73	36	26
ACM	13	4	2	2
IEEE Xplore	11	1	0	0
Total	104	78	38	28

**Table 7:** Main integration forms for each type of agile process (answer to RQ2).

Agile Process	Activity	Main Integration Forms
Scrum	Requirement Analysis Design Planification	Brainstorming session for user stories Iterative introduction of user requirements based on one or more functional and non-functional requirements Contextualization of the persona, design of the target group User feedback: evaluation, improvement, validation Development of the objectives by incremental iterations
XP	Requirements Analysis Refinement phase	Contextual research Integrate user stories with Personas-based design: empathize with the user to define an action for the problem Supervise the development of the prototype to ensure the use of the personas created Refactoring of personas (and even creation of new ones) with each change of requirements
DSDM	Requirements Analysis	User interviews Analyzing user stories in a design thinking session: creating sketches of personas Elaborate the solution design and validate it with users
FDD	Requirements Analysis	Questionnaires to users Clusters of users based on common preferences
Lean	Requirements Analysis	Questionnaire to know the user groups Clusters of users based on common preferences

## Appendix A

This Appendix shows the articles comprising the control group. Their titles, type of publication, and year of publication are listed in Table A1.

**Table A1:** Control group studies.

ID	Title	Authors	Type	Year
[26]	Adapting usability investigations for agile user-centered design	Sy, D.	Journal Article	2007
[27]	Probing an agile usability process	Wolkerstorfer, P., Tscheligi, M., Sefelin, R., Milchrahm, H., Hussain, Z., Lechner, M., and Shahzad, S.	Book Chapter	2008
[28]	Towards a framework for integrating agile development and user-centered design	Chamberlain, S., Sharp, H., and Maiden, N.	Conference	2006
[30]	User-centered design practices in Scrum development process: A distinctive advantage?	Anwar, S., Motla, Y. H., Siddiq, Y., Asghar, S., Hassan, M. S., and Khan, Z. I.	Conference	2014
[31]	Usability in agile software development: extending the interaction design process with personas approach	Haikara, J.	Conference	2007
[32]	Engineering m-learning using agile user-centered design	Rahim, W. A., Isa, W. M., Lokman, A. M., Taharim, N. F., and Wahid, N. D.	Conference	2014
[33]	Using persona with XP at LANDesk Software, an Avocent company Scrum	Broschinsky, D., and Baker, L.	Conference	2008
[34]	Current state of agile user-centered design: A survey	Hussain, Z., Slany, W., and Holzinger, A.	Conference	2009
[38]	Adopting user-centered design within an agile process: A Conversation	Hudson, W.	Journal Article	2003
[39]	User stories don’t help users: introducing persona stories	Hudson, W.	Journal Article	2013
[40]	Integration of eXtreme Programming and user-centered design: Lessons learned	Hussain, Z., Milchrahm, H., Shahzad, S., Slany, W., Tscheligi, M., and Wolkerstorfer, P.	Conference	2009
[41]	Two case studies of user experience design and agile development	Najafi, M., and Toyoshiba, L.	Conference	2008
[42]	Using agile software development methods to support human-centered design	Nakao, Y., Moriguchi, M., and Noda, H.	Journal Article	2014

## Appendix B

This Appendix shows the complete table of coverage, frequency, and weights of the words and sets of words analyzed in the control group articles. The calculation of the weights was obtained with the following formula:

((Word coverage)/(Maximum coverage) + (Word frequency)/(Maximum frequency))/2. (A1)

This way, the word that has the highest coverage and appears most often throughout the articles will have a weight of 1, and the rest will have a lower weight. The values for coverage, frequency, and weights for every word can be found in Table A2, and the complete table can be found at the following link: https://docs.google.com/spreadsheets/d/1OnsxdugBAPs_AN9Zfw8NOoYNbrKqL6yW9Vb7pVTuIj8/edit#gid=1696433063 (accessed on 18 September 2021).

**Table A2:** Complete list of words obtained from the selection process.

Words	Coverage (%)	Frequency	Weight
agile	100.00%	630	1
user	100.00%	613	0.987
usability	92.86%	578	0.923
product	100.00%	225	0.684
interaction	92.86%	148	0.585
study	92.86%	78	0.528
agile development	85.71%	94	0.505
method	92.86%	44	0.500
studies	85.71%	86	0.499
interaction design	85.71%	85	0.498
projects	85.71%	75	0.490
extreme programming	85.71%	53	0.472
ucd	64.29%	188	0.475
persona	71.43%	133	0.466
user experience	71.43%	123	0.457
user-centered design	78.57%	75	0.454
extreme	78.57%	70	0.450
hci	71.43%	114	0.450
xp	71.43%	111	0.448
agile software development	78.57%	55	0.438
Scrum	64.29%	90	0.395
integrating	71.43%	40	0.390
model	64.29%	63	0.373
agile method	57.14%	103	0.370
techniques	64.29%	46	0.359
ux	28.57%	271	0.364
sprint	57.14%	75	0.347
software engineering	57.14%	29	0.309
agile process	50.00%	22	0.268
interface design	50.00%	11	0.259
user stories	42.86%	32	0.240
lifecycle	42.86%	21	0.231
usability engineering	42.86%	20	0.231
agile project	42.86%	17	0.228
human-computer interaction	42.86%	15	0.227
human computer interaction	42.86%	10	0.222
conceptual	28.57%	22	0.161
user centered design	28.57%	15	0.155
user interaction	28.57%	4	0.146
usability method	21.43%	10	0.115
usability methods	21.43%	9	0.114
human-centered design	14.29%	13	0.082
software project	14.29%	9	0.079
software product	14.29%	3	0.074
user-centered design	14.29%	2	0.073
usability techniques	14.29%	2	0.073
usability technique	14.29%	2	0.073
user centered development	7.14%	5	0.040
user-centered development	7.14%	1	0.037
human centered design	7.14%	1	0.037
usability method	21.43%	10	0.115

## Appendix C

This Appendix shows the articles comprising the selected primary studies, listed in Table A3.

**Table A3:** List of selected primary studies.

Ref.	Title	Authors	Type	Year	Database
[27]	Probing an agile usability process	Wolkerstorfer P., Tscheligi M., Sefelin R., Milchrahm H., Hussain Z., Lechner M., Shahzad S.	Book Chapter	2008	Scopus
[31]	Usability in agile software development: extending the interaction design process with personas approach	Haikara, J.	Conference	2007	Scopus
[32]	Engineering m-learning using agile user-centered design	Rahim W.A., Isa W.M., Lokman A.M., Taharim N.F., Wahid N.D.	Conference	2014	Scopus
[33]	Using persona with XP at LANDesk software, an Avocent company Scrum	Broschinsky, D., and Baker, L.	Conference	2008	Scopus
[39]	User stories don’t help users: Introducing Persona stories	Hudson, W.	Journal Article	2013	Scopus
[40]	Integration of eXtreme programming and user-centered design: Lessons learned	Hussain Z., Milchrahm H., Shahzad S., Slany W., Tscheligi M., Wolkerstorfer P.	Conference	2009	Scopus
[48]	On the of use agile methodologies to re-design a Networks and Data Communications course	Vilchez-Sandoval J., Vasquez-Paragulla J., Llulluy-Nunez D.	Conference	2020	Scopus
[49]	Design thinking in software requirements: What techniques to use? A proposal for a recommendation tool	Parizi R., da Silva M.M., Couto I., Trindade K., Plautz M., Marczak S., Conte T., Candello H.	Conference	2020	Scopus
[50]	An inverted classroom experience: engaging students in architectural thinking for agile projects	Jane Cleland-Huang; Muhammad Ali Babar; Mehdi Mirakhorli	Conference	2014	ACM
[51]	Ideation: Generating as many ideas as possible	Quade S., Schlüter O.	Journal Article	2020	Scopus
[52]	AgileRE: Agile Requirements Management Tool	Gaikwad V., Joeg P., Joshi S.	Journal Article	2018	Scopus
[53]	Sprint: Agile specifications in Shockwave and Flash	Hakim J., Spitzer T., Armitage J.	Conference	2003	Scopus
[54]	Communication of design decisions and usability issues: A protocol based on Personas and Nielsen’s heuristics	Choma J., Zaina L.A.M., Beraldo D.	Conference	2015	Scopus
[55]	Enterprise software experience design: Journey and lessons	Sekar, B.	Conference	2017	Scopus
[56]	Persona Design in Participatory Agile Software Development	Dirks, S.	Conference	2008	Scopus
[57]	UserX story: Incorporating UX aspects into user stories elaboration	Choma J., Zaina L.A.M., Beraldo D.	Conference	2016	Scopus
[58]	Software creation workshop: A capstone course for business-oriented software engineering teaching	Paiva S.C., Carvalho D.B.F.	Conference	2018	Scopus
[59]	Flexible requirement development through user objectives in an Agile-UCD hybrid approach	Losada, B.	Conference	2018	Scopus
[60]	Applying agile methods and Personas to S-BPM	Forbrig P., Dittmar A.	Conference	2019	ACM
[61]	MEX experience boards: a set of agile tools for user experience design	Carvalho, C.	Journal Article	2010	ACM
[62]	Using Work System Design, User Stories and Emotional Goal Modeling for an mHealth System	Abdullah N., Grundy J., McIntosh J., How Y., Saharuddin S., Tat K., ShinYe E., Rastom A., Othman N.	Journal Article	2020	IEEE
[63]	Crowdfunding website design with lean product process framework	Perdana R.A., Suzianti A., Ardi R.	Conference	2017	Scopus
[64]	Information security application design: Understanding your users	Bhattarai R., Joyce G., Dutta S.	Journal Article	2016	Scopus
[65]	Top-down vs. bottom-up approaches to user segmentation: The best of both worlds	Mereu S., Newman M., Peterson M., Taylor E., White-Sustaita J., Yeats D.	Conference	2017	Scopus
[66]	Integrating design thinking into eXtreme Programming	Sohaib O., Solanki H., Dhaliwa N., Hussain W., Asif M.	Journal Article	2019	Scopus
[67]	Modelling agile requirements using context-based persona stories	Sedeño J., Schön E.-M., Torrecilla-Salinas C., Thomaschewski J., Escalona M.J., Mejias M.	Conference	2017	Scopus
[68]	Practical usability in XP software development processes	Hussain Z., Lechner M., Milchrahm H., Shahzad S., Slany W., Umgeher M., Vlk T., Köoffel C., Tscheligi M., Wolkerstorfer P.	Conference	2012	Scopus
[69]	Human stories—A new written technique in agile software requirements	Khanh N.T., Daengdej J., Arifin H.H.	Conference	2017	Scopus

## Appendix D

This Appendix shows the integration proposed in each individual study. The level of integration and the steps taken to integrate the Personas technique into an agile development can be seen in Table A4.

**Table A4:** Detailed integration method proposed by each selected primary study.

Study	Agile Process	Activity	Way of Integration	Integration Level	Integration Steps
[27]	XP	Requirements Analysis Planification and Design	Extreme Personas	Detailed	Start as the classic persona model. When the knowledge is updated, refactor the persona, or even create new ones if necessary.
[31]	XP	Requirements Analysis Planification and Design	Mobile-D (Extreme Personas)	Detailed	Exploration phase: research and modeling. Design: Requirements and framework definition. Development and testing: requirements refinement and prototyping.
[32]	XP	Requirements Analysis	Extreme Personas	Detailed	Contextual research. Consolidation in Personas. Interview with users to evaluate satisfaction with the prototype.
[33]	XP	Requirements Analysis Planification and Design	Alan Cooper on XP + contextual design	Detailed	Ethnographic research. Asynchronous research to gather info on specific characteristics without affecting the speed of the project. Validating personas with end-users. Monitoring of developers to confirm that they use personas.
[39]	Scrum	Requirements Analysis	Minimal personas, persona stories	Detailed	Short description and motivation for each persona. One page per persona: one side for utilities and preferences and one side for likes and personality. Persona stories: how a particular persona (not the specific users) does the task.
[40]	XP	Requirements Analysis	Lightweight Personas	Detailed	Contextual research. Creates personas based on initial studies. Refactor when the study suggests changes in requirements.
[48]	DSDM	Requirements Analysis	Sketch and user interview	Generic	
[49]	DSDM	Requirements Analysis	User-centered creative thinking, priority specification during prototyping	Detailed	Design-thinking session for user story analysis. Requirement specification activity. Interview with users for validation.
[50]	DSDM	Requirements Analysis Planification and Design	Architecturally Significant Persona	Detailed	Analyze user stories. Create personas and assign them user stories. Elaborate solution design and validate with users.
[51]	Scrum	Planification and Design	Creative session between requisite analysis and planification	Detailed	Brainstorming session for the creation of user stories. Grouping similar ideas in one persona.
[52]	Scrum	Requirements Analysis Planification and Design	AgileRE Tool	Detailed	Template to create persona with auto-complete sentences. Create epics and user stories. When the user is satisfied, mark the story as solved.
[53]	Scrum	Requirements Analysis	Creative session with students	Generic	
[54]	Scrum	Requirements Analysis	Nielsen heuristics	Detailed	Select artifacts and hypotheses of relevant personas. Associate tasks to each persona.
[55]	Scrum	Requirements Analysis	Mental maps to connect Personas	Generic	
[56]	Scrum	Requirements Analysis	4 steps to create personas with physical or mental limitations	Detailed	Introduction of user requirements iteratively. Contextualization of the persona, design of the target group. Concretization. Development based on user stories. Feedback: evaluation, improvement, testing.
[57]	Scrum	Requirements Analysis	User XStories	Generic	
[58]	Scrum	Project Definition	Study based on personas along the project definition	Generic	
[59]	Scrum	Requirements Analysis	User objectives on incremental iterations	Detailed	Specification of requirements at the interface and navigation level. Presentation of the user objectives from one or several functional and non-functional requirements each, to obtain high fidelity prototypes. Validation of the functionality of the user objectives with the Personas technique.
[60]	FDD	Requirements Analysis Planification and Design	Combination of Personas and use cases	Detailed	Combine use case actors with subjects in personas. Use information from personas in S-BPM interaction diagrams: sub-stories of subjects for behavioral models. Refine user stories by abstracting patterns and assigning these small sub-stories to concrete subjects.
[61]	FSS	Requirements Analysis	Light personas—MEX boards	Generic	
[62]	FDD	Requirements Analysis Planification and Design	Questionnaire to patientsInterviews with emotional analysis experts	Generic	
[63]	Lean	Problem Definition	Questionnaire during the problem definition	Detailed	Questionnaire to determine the user groups.
[64]	Lean	Requirements Analysis	Proto-Persona method	Detailed	Development of the persona. Knowing the users. Refining the persona.
[65]	Lean	Requirements Analysis Planification and Design	User clusterization according to behaviors and preferences	Detailed	Contextual research with interviews. Grouping the results into clusters of Personas based on preferences and behaviors. Design for the patterns found in each cluster.
[66]	XP	Requirements Analysis Planification and Design	Design Thinking	Detailed	Integrate user stories with persona-based design: empathize with the user and define an action to the problem based on it. Multidisciplinary team for collaboration and creativity. Prototype development. UCD and user acceptance. Usability testing during agile development by the user.
[67]	XP	Requirements Analysis	Persona story context metamodel	Detailed	Create the context of each persona. Describe the interaction between several personas in order to reduce the processing time of each use case. Represent the interaction between personas in a class diagram.
[68]	XP	Requirements Analysis Refinement phase (changes among iterations)	Extreme Personas	Detailed	Personas are modeled based on the preliminary user groups. Personas are refactored each time new information appears suggesting changes. Creation of new personas if the current ones do not meet the new needs.
[69]	XP	Requirements Analysis	Human story as a combination of User Story and Persona Story	Detailed	Give the persona a name. Mind map design to connect what the persona wants and how they want it. Use colors to highlight what is important. Understand the user before developing for them. Importance of story writing. Evaluate the result with agile requirements quality checklists.
